# Selection and Conservation of Gametes and Embryos for Improving Assisted Reproductive Technologies

**DOI:** 10.3390/ani13172761

**Published:** 2023-08-30

**Authors:** Andrés Gambini, Isabel Ortiz, Manuel Hidalgo, Jesús Dorado

**Affiliations:** 1 School of Agriculture and Food Sustainability, The University of Queensland, Gatton, QL 4343, Australia; 2 School of Veterinary Science, The University of Queensland, Gatton, QL 4343, Australia; 3 Veterinary Reproduction Group, Department of Medicine and Animal Surgery, Faculty of Veterinary Medicine, University of Cordoba, 14014 Cordoba, Spain; iortiz@uco.es (I.O.); mhidalgo@uco.es (M.H.)

We are delighted to present this Special Issue, which is dedicated to the paramount subject of gametes and embryo selection and conservation for improving Assisted Reproductive Technologies (ARTs). Breeding technologies have emerged as a transformative force in both human fertility treatments as well as animal production and conservation efforts. This Special Issue brings together a collection of groundbreaking research that delves into various aspects of gamete and embryo selection, cryopreservation, in vitro maturation, and oocyte activation, shedding light on significant advancements and challenges in the field of ART for animal production and preservation.

The ability to manipulate and preserve gametes and embryos has paved the way for enhancing reproductive efficiency, infertility treatments, genetic selection, and breeding programs in diverse animal species. In agricultural settings, ARTs play a critical role in optimizing livestock and aquaculture production, enabling the propagation of genetically superior individuals and improving overall herd or stock quality. Moreover, the conservation of endangered species has become an urgent global concern. ARTs have emerged as powerful tools for safeguarding genetic diversity and rescuing species on the brink of extinction. The cryopreservation of gametes and embryos has opened new avenues for preserving the genetic heritage of threatened wildlife, offering hope for restoring populations and facilitating successful breeding and reintroduction programs.

The manuscripts presented in this Special Issue represent the forefront of the research into assisted reproduction for animal production and preservation. Each article contributes to our understanding of gamete and embryo selection processes and their direct implications for improving ART outcomes. The studies presented here cover a wide array of species, including livestock, canines, fish, and wildlife, underscoring the broad applicability and significance of ART across diverse taxonomic groups.

Selionova et al., 2023 [1], delves into the cryo-resistance of Charollais sheep embryos, investigating the influence of developmental stage and freezing methods on embryo survival. This study sheds light on the optimal embryo development stages and the most effective cryopreservation techniques for Charollais sheep. The findings have the potential to optimize cryopreservation protocols for ovine species, improving the preservation of valuable genetic resources and facilitating artificial breeding programs.

Astudillo et al., 2023 [2], explores the intriguing phenomenon of poly-ovular follicles in canines and their impact on the in vitro maturation of oocytes. This study offers insights into the unique meiotic development of canine oocytes and its implications for canine reproductive technologies. The results provide valuable knowledge for improving in vitro culture strategies and reproductive outcomes in companion animal breeding, contributing to population management efforts.

An essential aspect of this Special Issue centers on sperm cryopreservation in Sorubim cuspicaudus, a commercially significant fish species. The study reported by Atencio-García et al., 2023 [3], meticulously evaluates the effects of cryopreservation on sperm quality, addressing crucial factors that influence fertilizing capacity. The outcomes will inform the aquaculture industry’s objective of optimizing sperm cryopreservation protocols, thereby improving reproductive success and supporting sustainable aquaculture practices.

In addition, a compelling study reported by Cabeza et al., 2022 [4], uncovers the role of zinc chelation in oocyte activation and embryo development in bovine and porcine species. Understanding these activation mechanisms is paramount for refining ART protocols, offering potential benefits for both livestock production and biomedical research communities. The findings of this study contribute to enhancing our knowledge of oocyte activation, a crucial step in successful ARTs, and it is the first report on producing blastocyst by means of zinc chelation in the bovine species.

Lastly, a comprehensive review article by Huijsmans et al., 2023 [5], critically analyzes the postmortem collection of gametes for conserving endangered mammals. By synthesizing existing knowledge and discussing challenges and opportunities, the review emphasizes the importance of refining protocols tailored to different species, ultimately contributing to the conservation of endangered wildlife. This review article serves as a valuable resource for conservation biologists and researchers working to preserve the genetic diversity of threatened species.

As editors of this Special Issue, we extend our sincere gratitude to all the authors, reviewers, and editorial staff whose contributions have made this collection of articles possible. We are confident that this compilation will serve as an invaluable resource for researchers, practitioners, and policymakers, fostering innovation and collaboration to further improve Assisted Reproductive Technologies in animal production and conservation.

We hope that the insights shared in this Special Issue will propel the field forward, addressing challenges and opening new avenues for enhancing reproductive efficiency, genetic diversity, and sustainability in animal production.
